# Extinction risk of soil biota

**DOI:** 10.1038/ncomms9862

**Published:** 2015-11-23

**Authors:** Stavros D. Veresoglou, John M. Halley, Matthias C. Rillig

**Affiliations:** 1 Freie Universität Berlin, Institut für Biologie, Plant Ecology, Altensteinstrasse 6, D-14195 Berlin, Germany; 2 Berlin-Brandenburg Institute of Advanced Biodiversity Research (BBIB), D-14195 Berlin, Germany; 3 Department of Biological Applications and Technology, University of Ioannina, 45110 Ioannina, Greece

## Abstract

No species lives on earth forever. Knowing when and why species go extinct is crucial for a complete understanding of the consequences of anthropogenic activity, and its impact on ecosystem functioning. Even though soil biota play a key role in maintaining the functioning of ecosystems, the vast majority of existing studies focus on aboveground organisms. Many questions about the fate of belowground organisms remain open, so the combined effort of theorists and applied ecologists is needed in the ongoing development of soil extinction ecology.

## Abstract (web summary)

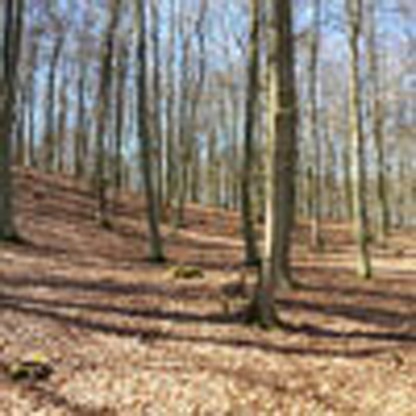
Belowground soil biota play key roles in maintaining proper ecosystem functioning, but studies on their extinction ecology are sparse. Here, Veresoglou *et al*. review the risks to soil biota posed by global change, and highlight the technical challenges involved in identifying extinction events.

The belowground compartment of ecosystems harbours a tremendous amount of global biodiversity. While our ecological understanding of belowground organisms is limited, it has become evident that these organisms may be less functionally redundant than originally thought^1^,^2^. Many arguments for preserving biodiversity line up with our moral obligation to conserve nature, but it is increasingly apparent that extinctions could also compromise our ability to receive ecosystem services and this could become the basis for intensifying conservation^3^. For belowground organisms, as the aesthetic and moral arguments are less intuitive, the need to avoid species loss to sustain ecosystem functioning is especially pertinent. However, for any form of conservation we need a basic understanding of the susceptibility to extinction. For soil organisms this issue so far has been dealt with only cursorily compared with the aboveground world^4^. Our review of the state of soil extinction ecology reveals a worrying paucity of existing information. However, the challenge of understanding and modelling belowground extinction also opens up a rich frontier of opportunities for future research.

## Modelling extinction

**Figure 1:**
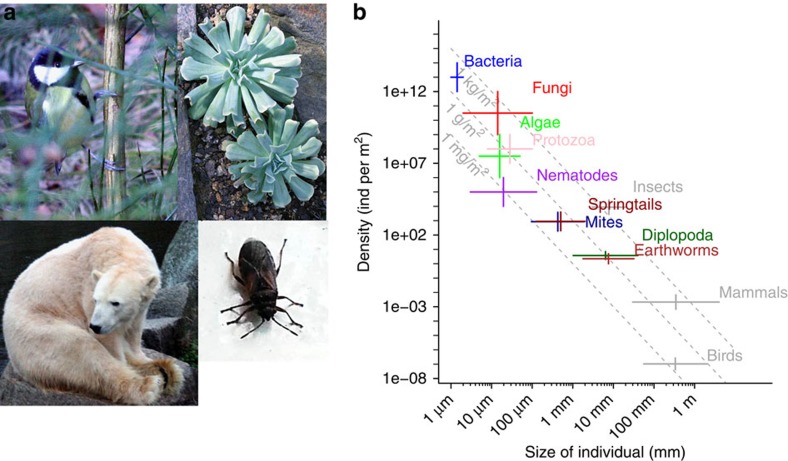
Size–density relationships for below- and above-ground organisms. (**a**) Representative images of organisms that belong in the four groups that have been studied so far in extinction ecology: birds, plants, mammals and insects; (**b**) size–density relationships in representative belowground (in colour) and aboveground taxa (in grey). Belowground taxa size estimates are according to the study by Swift *et al*.^83^ Size estimates of aboveground taxa were obtained following searches for the size of organisms with extreme sizes. Belowground taxa density estimates were retrieved from a synthesis of data from ‘European Crop Protection Association. Soil Biodiversity and Agriculture (2010) (http://www.ecpa.eu/files/gavin/soil_bio_and_ag_012_web.pdf)'. Densities for aboveground organisms originated from projections of global population estimates for taxonomic groups found in ‘Tomasik B. (2014) How Many Wild Animals Are There? (http://reducing-suffering.org/how-many-wild-animals-are-there/)'. Note that even without consideration of microbial taxa the variability for belowground organisms exceeds that of the aboveground taxa so far studied in extinction ecology. Image credits: S.D.V.

Extinction ecology studies the conditions under which species go extinct locally or globally (Box 1). The vast majority of the literature, to date, focuses on the extinction dynamics of macroscopic aboveground organisms^4^ (Fig. 1a). Most organisms that have inhabited the earth are already extinct, with extinction events often happening simultaneously in what are known as mass extinctions. There have been five mass extinctions so far and an ongoing sixth mass extinction is argued to be currently taking place^5^.

While extinctions can sometimes be tracked down to environmental stochasticity alone, the two most important recognized causes of extinction today are the loss of habitat and the introduction of invasive species^6^,^7^. According to the widely recognized Arrhenius species–area relationship, the biodiversity that can be sustained in a finite region increases as a power of the area of that region^6^. Because species–area relationships link habitat size to diversity such relationships have been frequently used to forecast extinction due to habitat loss. There are several approaches available to model extinction. One way to model the extinction risk of an organism is through carrying out a population viability analysis, combining information on the demographics of the population and its ecological niche structure^8^. An alternative way is to ignore the ecological niche of the organism and assess extinction risk based on ecological drift (that is, demographic stochasticity) alone; this can be done through implementing a neutral model^9^. Niche and neutral approaches to extinction are important from a modelling perspective and there have been calls from extinction ecologists to develop further extinction models that combine the two approaches^10^.

In the literature on extinction ecology, reports pertaining to belowground organisms are alarmingly sparse^11^ (see Box 2). Moreover, much of the existing evidence originates from relatively artificial experiments^12^,^13^. For example, Gonzalez and Chaneton^12^ studied the effect of habitat fragmentation on microarthropod dynamics in an artificial moss system. While microarthropods are the group of soil biota that are preferably used in controlled systems to study extinction dynamics (for example, ref. 14), considerable literature on habitat fragmentation in controlled experiments also exists with regards to soil fungal decomposers. While microcosm studies may result in considerable discussion^13^,^15^,^16^,^17^ and provide unique insights on extinction ecology, the results cannot always be scaled up to be informative about the extinction susceptibility in the field. Several studies in the literature have addressed diversity shifts of microbial and soil communities in response to global change drivers^18^,^19^ but no studies have yet focused on the connection between biodiversity shift and extinctions. There have thus been only few *in situ* studies on the potential of belowground organisms to become locally extinct, while no studies have addressed global extinctions. For example, Berglund *et al*.^11^ found evidence that species–area relationships were considerably more accurate for wood-inhabiting fungi than for lichens, a result that was attributed to a prolonged relaxation time before any extinctions could be observed in the case of lichens. Jönsson *et al*.^20^ monitored local colonization and extinction dynamics of wood-inhabiting fungi over 6 years, discovering that the key predictors of local extinction dynamics were site quality and connectivity. Local or global extinctions of soil biota thus represent a huge blind spot for reasons that are most likely due to the very special nature of the soil habitat.

## Belowground challenges for extinction ecologists

### Microhabitat complexity

Soil represents a challenging habitat to study. It is an intricately structured three-dimensional habitat which, due to the small size of the soil organisms, is subject to considerably more layering than even the most extensively vertically layered aboveground habitats such as tropical forests^21^. Considering the fabric of belowground habitats in extinction ecology is an exceptionally important point. The nature and structure of soil also gives rise to unparalleled microhabitat complexity with numerous physiochemical gradients, for example, aerobic and anaerobic microhabitats can occur directly adjacent to each other (that is, within a few dozen micrometres) due to diffusion limitation in pore networks^22^. The resulting high complexity of the soil landscape could render soil biota considerably more resistant to change than aboveground organisms, as suggested in the landscape-moderated insurance hypothesis^23^. In addition, soils may be insulated against many drivers of climate change, including drought, warming and extreme events. For example, natural CO_2_ levels in the soil atmosphere are much higher than in the air above, because of soil biological activity (including root and microbial respiration) coupled with gas diffusion limitation. Also, soil offers temperature insulation, and at the microscale it may be partially insulated against drought events through capillary water reserves. By contrast, in the short-term soil communities may be particularly susceptible to anthropogenic stressors such as ploughing. Thus, the features of the soil (micro-)habitat alone impose a range of distinct considerations for the ecologist delving into belowground extinction susceptibility.

### Extinction models

**Figure 2:**
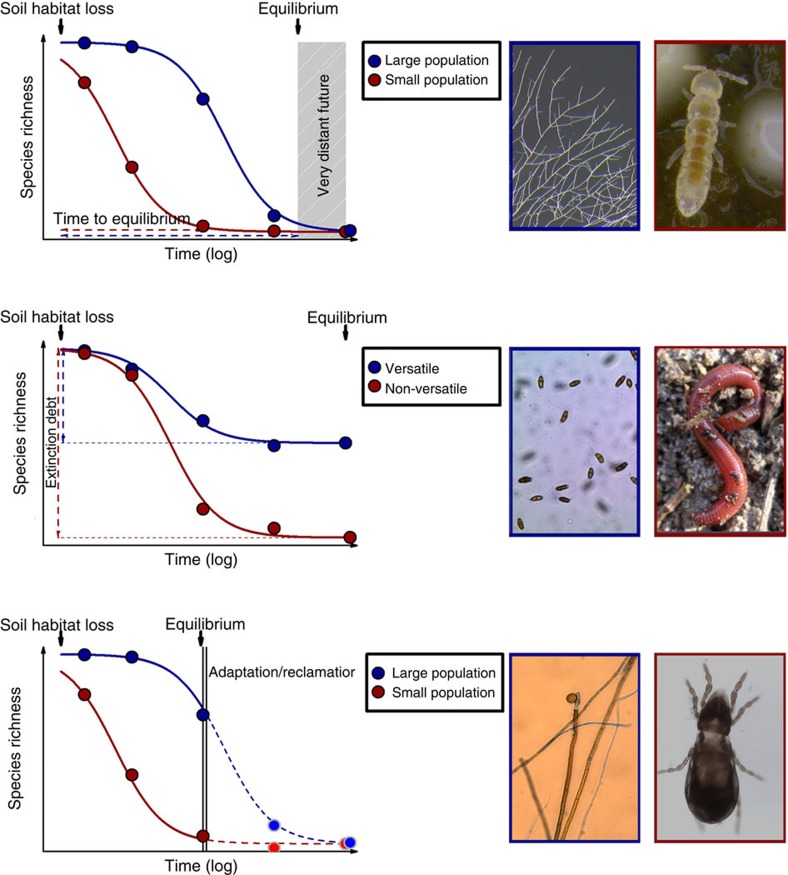
Particularities of extinction susceptibility for soil biota. Differential extinction susceptibility as influenced by three particularities of belowground food webs, (**a**) extreme population sizes—population sizes for belowground organisms may exceed considerably the minimal viable population thresholds, or alternatively for asexual organisms the minimal viable population could coincide with a single individual; (**b**) physiological versatility—definitions of habitat loss for belowground organisms differ considerably from those for aboveground organisms and physiologically versatile organisms may suffer less from changes in their habitat; (**c**) high adaptation potential^1^,^84^ that is particularly relevant to the microbial taxa (in the panel population size factors that could affect adaptation speed were not considered but could have exacerbated differences). Representative examples of each group of belowground organisms are presented in the images on the right of each panel. For modelling extinction debt a hyperbolic extinction trajectory over time was assumed. Figure design based on the study by Kuussaari *et al*.^35^ Image credits: top left panel—Anika Lehmann; middle and bottom left panels—Diana Andrade; top and bottom right panels—Stefanie Maaß; Middle right panel—Karoline Weißhuhn. Reproduced with permission from the authors.

Extinction models for belowground organisms are currently unavailable; thus, soil ecologists at the moment have no choice but to generalize the predictions for aboveground biota to their system^24^,^25^. Yet, the ecology of belowground biota differs considerably from that of the aboveground organisms that have so far been considered in extinction models (Fig. 1)—at least with regard to their niche structure which we consider in the following. The most conspicuous difference relates to body size variability which, even without considering belowground microbes, is several orders of magnitude larger in soils (Fig. 1b). Unlike their aboveground relatives, microbial organisms in soil also represent the base trophic level of their food webs^26^ and are responsible for overall ecosystem functioning. While aboveground ecologists can afford to overlook microbes in community models, this is not possible in soil ecology. The small size of the organisms in soil also translates to greater population sizes than for most currently considered aboveground taxa (Fig. 1b). Even though the Baas–Becking postulate, an intensely debated tenet according to which ‘everything is everywhere but the environment selects', has been convincingly rejected for many groups of microbial organisms^27^ (but see ref. 28), microbes are still believed to generally occupy extensive geographic ranges relative to their size^29^. Extensive biogeographical distribution may lead to large populations of soil organisms, which from an extinction ecology perspective, are important because these are subject to very long extinction trajectories following habitat loss (Fig. 2a). An additional difference is the fact that most groups of belowground taxa can propagate asexually (for example, bacteria, fungal mitospores) often in addition to having sexual reproduction. Asexual organisms can be subject to population dynamic forces quite different from those of sexually reproducing species. Especially relevant for extinction ecology is the fact that for asexual organisms, a single individual can be a viable population and also many properties important for sexual reproduction such as the sex ratio do not apply. Furthermore, specifically for microbial taxa, the fact that they can be extremely physiologically and functionally versatile represents an additional facet of differentiation from existing aboveground extinction ecology. For example, microbes possess an arsenal of cryptic genes (that is, phenotypically silent genes) that allow them to cope with environmentally adverse conditions for extensive periods^30^. Soil microbial taxa can also concurrently or consecutively pursue functionally very divergent lifestyles such as those of a root-endophytic parasite and a decomposer of soil organic matter (many soil fungi^31^) or of a nitrogen fixer and a free-living symbiont (bacteria of the genus *Azospirillum*^32^). Still other belowground taxa may possess ecophysiolocal traits comparable to macroscopic species that live aboveground. These pronounced ecological dissimilarities can affect the relative susceptibility of different taxa to habitat changes and need to be accounted for in extinction models (Fig. 2b,c). Finally, many microbes possess the ability to form resting structures for extensive periods of time, even exceeding those known from seed banks of plants^33^. This coupled with the high prevalence of conditionally rare microbial taxa observed in soil^34^ result in highly complex population dynamics that are exceptionally tricky to either monitor or model.

### Temporal scales of extinction

The fact that extinctions do not happen instantaneously adds an additional layer of challenges to belowground extinction ecologists. Extinction debt^35^ reflects the delay in diversity loss after habitat contraction before the species richness reaches the level predicted by the species–area relationship. The relaxation time of this process has been linked to habitat size^36^. As argued in the previous paragraph, in soil we expect this relaxation time to be disproportionally high for a subset of organisms due to large population sizes and asexual propagation (Fig. 2a). This could very well mean that humanity has not yet witnessed the full consequences of soil habitat destruction, even given the already well-appreciated adverse effects of agriculture, erosion and desertification. On the other hand, if soil habitat could be restored, impending extinctions could be forestalled. Preserving a handful of suitably interconnected soil habitats at an adequate spatial scale might be adequate. Placing susceptibility to extinction of belowground organisms in an extinction-debt framework makes apparent that pronounced differences across organismal groups are likely, much larger than those that have so far been considered in aboveground extinction models (Fig. 2). We need to also be very careful about extrapolating or applying concepts of extinction dynamics to microbes: here we enter exciting, but unchartered territory.

**Figure 3:**
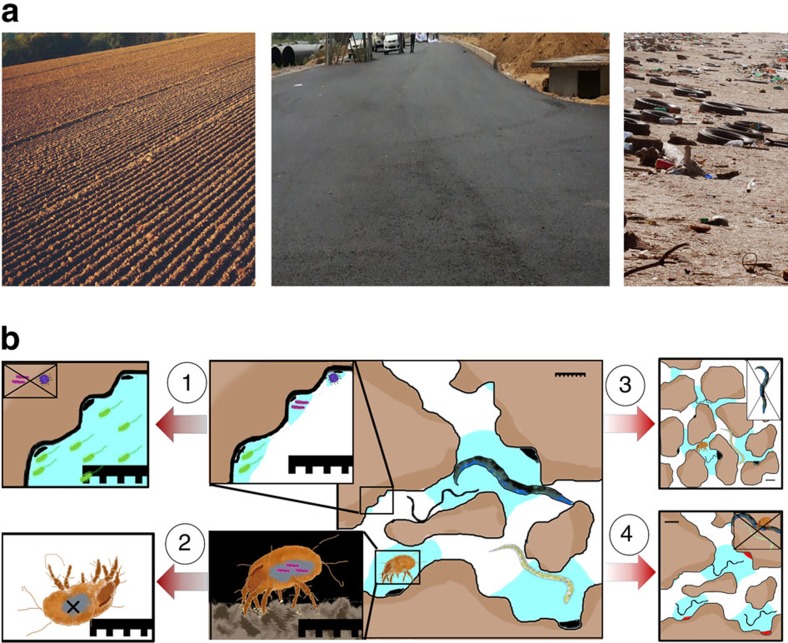
Macro- and micro-scale perspectives of habitat loss belowground. (**a**) A more anthropocentric perspective of what represents habitat loss for belowground ecosystems: tillage, urbanization, pollution; (**b**) from a soil biota perspective the drivers of extinctions can be localized, however, at a more intricate, microscopic level. At this microscale level, the effects of (1) water availability and aeration; (2) host extinction; (3) loss of soil structure; (4) declines in carbon substrate availability are depicted within a soil habitat. The three macroscopic examples of habitat loss are linked to various effects at the microscopic level. For instance tillage can compromise aeration, lead to extinction of arthropod hosts and impair soil aggregation; urbanization other than impacting arthropod hosts can have pronounced effects on substrate availability; and soil pollution can affect carbon substrate availability. Note scale in images. Image credits (Author, ‘description', year, modifications (license)): left panel—We El, ‘Beploegd veld', cropped, 2005 (CC BY-SA 3.0), central panel—Baba Ovian, ‘Dwarka Expressway', cropped, 2013 (CC BY-SA 3.0), right panel—Nils Ally, ‘Litter', cropped, 2010 (CC BY 3.0). Source: Wikipedia. Used according to the terms of a GNU Free Documentation License.

It is unequivocal that at temporal scales exceeding single geological periods, soil organisms do go extinct; a number of organisms that are known to have existed several millions of years ago are only distantly related to existing species^37^. For a subset of soil biota persistence is tightly linked to hosts such as plants, through symbiosis, so extinction risk can likely be tied strongly to the demographics of these hosts (Fig. 3b). Another subset of soil biota such as soil decomposers that may not be host specific (but see ref. 38) but have evolved a high affinity to specific compounds (for example, white-rot fungi and lignin) could be susceptible to high-impact events that can modify the availability of their substrate. For example, microbes apparently responded strongly to the Permian and the Triassic mass extinction events^39^ and they could respond similarly to the high ongoing extinction rates currently observed^5^. On the other hand, the hotspots of extinction for belowground organisms could be very different from those for aboveground organisms. This is because there is only a loose relationship between aboveground and belowground diversity. For example Tedersoo *et al*.^40^ showed that the plant/soil fungus richness ratio declines as a negative exponential from equator to pole, suggesting a decoupling between aboveground and soil diversities. In another study, Ramirez *et al*.^41^ compared local versus global diversity patterns of soil bacteria and fungi, finding comparable gamma diversity estimates for the two kinds of data sets.

### Niche versus neutral processes

Up to this point we have highlighted the specific features of soil and its inhabitants. Another approach for modelling extinction is to ignore the niche structure of belowground communities and implement a neutral approach. Using a neutral approach could be advantageous because many of the challenges related to characterizing ecological niches of soil biota could be bypassed. However, in the case of a neutral model, there are some important considerations. On the one hand, the trophic structure of belowground food webs is poorly resolved and for some organisms it is hard to identify the trophic level to which they belong, in part due to widespread omnivory^26^. This is important because neutral theories are most suitable for organisms within a single trophic level. On the other hand defining the concept of species for microbes is not as straightforward as it tends to be for macroorganisms, because these definitions rely on assignments of dissimilarity in sequence data. Up to now neutral models have relied on robust definitions of species and this could represent a major issue. Moreover, even with reliable quantification of soil taxa abundances, fitting neutral models could be problematic because of the sheer size of belowground communities. Large population sizes imply low sensitivity of the organisms involved in stochastic events, which represent the driving forces of extinctions in neutral models^9^ but also spell difficulties in assessing neutral theory parameters based on observations within scientifically feasible monitoring timespans (Fig. 2a).

As stated above, our ignorance of how widespread functional redundancy may be in soil represents one of the important issues that soil extinction ecologists face, because this determines how imperative soil conservation measures are for securing ecosystem services. A hypothetical scenario in which organisms go extinct at low frequencies and where there is a high functional redundancy across organisms would imply a low priority of conservation efforts compared with a scenario where extinctions occur at a high rate and there is little to no functional redundancy. Moreover, the extent of potential functional redundancy differs across ecosystem functions with some functions being performed by specialized organisms, such as ammonia oxidation, and others by a broader suite of soil biota, such as denitrification. In an influential study addressing functional redundancy in plant communities, Isbell *et al*.^42^ showed that broadening the study of the functional role of plant species to multiple growth seasons and environmental conditions resulted in no plant species being identified as functionally redundant. It is very likely that functional redundancy in soils could behave similarly. Unravelling the degree of functional redundancy in soil could represent a huge step in explaining microbial community dynamics over time^28^. It could also hint at the relative importance of niche versus neutral dynamics in soils. If redundancy in soil is common then it is likely that a lot of functionally ‘equivalent' taxa exist and that stochastic drivers are of particular importance in driving soil communities; these can be better studied through neutral models. If, however, there is little to no redundancy across soil organisms then the challenge is in attaining sufficiently sophisticated extinction models that consider components of the niche structure of the soil biota, which can be used for predictive purposes.

## Extinction risk factors for soil organisms

### Habitat loss and global change

The extinction ecology for the majority of belowground organisms is thus likely to be quite different from that of the macroorganisms that have been considered so far in the literature. To gain informative insights on extinction susceptibility it is important to first compare the drivers of prospective extinctions belowground with those that have been studied so far in extinction ecology. This is the purpose of the next section.

The best documented drivers of extinctions are habitat loss and fragmentation. For aboveground organisms, habitat loss relates in one way or another to the removal of the autotrophs that dominate an area. For belowground organisms, a radically different perspective of habitat loss is required in many cases. In some situations, the perspective is rather similar; for example, urbanization perhaps provides one of the most intuitive examples of belowground habitat loss. Urbanization, through the eradication of autotrophic organisms and fragmentation of the soil surface via cementing, mirrors definitions of habitat loss that are routinely used in the ecology of macroorganisms (Fig. 3b). Pavao-Zuckerman and Coleman^43^, for example, have shown that the abundance of predatory nematodes declines following urbanization. Global change can also represent a driver of habitat loss comparable to the way this occurs for macroorganisms (Fig. 3a). Extreme drought events have been shown to strongly impact belowground habitats^44^ and mostly have effects analogous to the aboveground compartment of ecosystems. However, climate change can also have indirect, substantially more intricate and less intuitive effects on soil properties that must be considered habitat loss. Niklaus *et al*.^45^ have shown that elevated atmospheric CO_2_ indirectly led to reduced soil aggregate and pore sizes, which in turn likely led to an extinction of large-diameter nematodes because of habitat loss: simply the pore space these organisms require for movement was no longer available. Moreover a range of anthropogenic activities can exert long-lasting effects on soil biota that could lead to local extinctions. Soil tillage and fertilization are two characteristic examples. The former may cause a deterioration of soil structure, because soil aggregates are destroyed^22^, whereas the latter may result in competitive exclusion of soil taxa with an oligotrophic life-strategy. Both these drivers can exert a constant selection pressure on belowground organisms^46^ and consequently represent causes of habitat loss for a subset of the community. Another example is irrigation, which has been linked to local extinctions of earthworm taxa^47^. It is hard to encapsulate the extent to which each of these drivers can lead to the loss of belowground diversity but the scientific community needs to work on this. It has been shown that a number of bacterial^48^ and fungal^49^ taxa are particularly susceptible to intensification of land use. Specific examples are presented in Box 2. Many of these susceptible taxa are of major functional importance for both belowground and aboveground food webs^50^.

### Competition and specialization

Another risk factor for soil biota could arise from microbial invasions. While microbes have received comparatively limited attention in the invasion literature, increased worldwide human travel is expected to increase unintentional invasions^51^,^52^. Invasions of belowground microbes, compared with those of macroscopic organisms, would be considerably more difficult to control due to the small size of the organisms. The extent to which principles that have been proposed for aboveground organisms could be valid for soil biota is unknown. For example, for macroorganisms it has been argued that invasive predators can increase the extinction risk of native biota considerably more than invasion of organisms with exceptional competitive ability^7^. If this principle applies for belowground taxa it would be invasions of predatory microarthropods and protists that require attention rather than bacterial or fungal invasions. We also do not know whether and to what extent soil communities that are easily invaded may be more prone to extinctions than invasion-resistant communities^53^. Although it is challenging to assess invasion resistance for belowground communities, there is evidence that at least for microbes invasion resistance can be related to diversity^54^. It could hence be the case that belowground biota that possess adaptations to specific low-diversity habitats are more susceptible to extinction than other biota.

While many soil fauna are omnivores with little apparent specialization, a subset of soil animals exhibit feeding specialization^26^. A pervasive idea in extinction ecology is that highly specialized species may be more prone to extinction than generalists because their persistence additionally depends on the persistence of their prey^55^. Soil animals might thus be considerably more susceptible to extinctions than soil microbes, and given that litter decomposition hinges to a large degree on soil animal activity this can have important implications for soil ecosystems^56^. Following a similar logic, endosymbiotic soil microbes, which are vertically transmitted, and other obligate symbionts could also be more susceptible to extinction than free-living microbes.

A final hypothetical risk factor for belowground organisms could be genetically modified organisms (GMOs). GMOs could threaten soil biota in two ways. One concerns the accidental release of GM belowground taxa to the environment, which may have consequences similar to those discussed with other invasions. The second concern relates to non-specific effects of using GM crops. There have been a number of studies that have addressed this topic, which suggest overall effects on belowground diversity are perhaps not as severe as originally feared, and no study on GMOs has so far demonstrated soil biota extinctions^57^. Yet, given the high host specificity of some belowground biota, specific effects on individual taxa are possible as has been shown for some better-studied aboveground taxa^58^.

## A pragmatic approach to soil biodiversity conservation

### Beyond the black box

Above we argued that the approach to soil conservation could be more pragmatic than that for macroorganisms. Is sustaining current ecosystem functions the sole reason to take seriously the state of the soil^42^? Soil carries many more benefits for humanity: soil biodiversity losses could lead to release of soil-based opportunistic human pathogens^59^, undermine our ability to combat the growing resistance of clinical microbes^60^,^61^ and compromise the resilience of soil ecosystems in the face of global environmental change^62^ with serious consequences for food production in a changing world. Considering biodiversity adds another layer of good reasons to monitor what is happening.

Working with belowground organisms to either assess extinction susceptibility or extinction status is currently not easy, and may never be. Until relatively recently, soil biodiversity was mostly regarded as a spatially undifferentiated black box^27^,^63^ and only now have we begun to appreciate the immense diversity of soil microbial habitats^64^. Recent years have seen a revolution in terms of molecular techniques, and now dedicated deep-sequencing projects reveal that our routine sequencing analyses produce relatively blurry pictures of the organisms present. These are not yet directly suitable for monitoring extinction dynamics^65^, but techniques and bioinformatic analysis pipelines are continually improving. There has been growing realization, also in terms of funders, that monitoring belowground communities routinely necessitates costly molecular analyses, and that extensive assays of microbial taxa are needed for quality analysis of the dynamics of rare microbial taxa.

Despite marked progress over the last few decades, currently soil ecology still lags far behind aboveground ecology^66^, and our knowledge of the world belowground is comparatively limited. What kinds of tools and methods are going to be needed to make progress? A way forward could relate to intensifying monitoring of microbial population dynamics, at small spatial scales and with adequate sequencing effort, to determine local extinction and recolonization rates for taxa of interest. Such results can be integrated with estimates of global microbial population sizes, which are now becoming available within the flourishing field of microbial biogeography^67^, in the form of basal data for microbial extinction models. The accuracy of such early extinction models could be further improved through consideration of ecological interaction network topologies. Secondary extinctions are expected to be important in nature both in aboveground and belowground food webs^68^; they affect extinction risk and, assuming that the network topology can be accurately conceptualized, they could be predicted^69^. Belowground extinction models could thus benefit greatly from prospective progress in ecological network theory^70^. Establishing linkages between population dynamics of belowground organisms and the fractal geometry of the soil could also facilitate modelling extinction dynamics in soil^71^. Finally, due to the inherent particularities of the definition of ‘habitat loss' in soil, consideration of the current habitat status/size may not be as important as its history^72^. Belowground extinction models could thus be improved by including parameters relevant to the frequencies of disturbance events such as fertilization pulses, clearcut-logging and tillage.

### Conservation prioritization

Given that belowground diversity may indeed be declining, identifying taxa of special conservation interest should be a priority in emerging management schemes. A number of existing belowground conservation initiatives appear to prioritize conservation of ecosystem processes over conservation of species^73^. The focus on ecosystem processes is reasonable but requires a good understanding of how belowground diversity links to ecosystem functioning, which is currently unavailable. We suggest that to the extent possible, until we attain an adequate understanding of brown food webs, conservation initiatives should also consider conserving belowground taxa of high ecological interest. Keystone belowground taxa could be identified based on the properties of their respective interaction networks^69^. Simulations with microbial interaction networks have revealed that the predictive accuracy of identifying keystone species based on the structure of co-occurrence networks alone exceeds 80%, as keystone species appear to share a number of properties such as a high mean degree, a low betweenness centrality, a high closeness centrality and high transitivity^74^. The prediction accuracy could further improve in future through our better understanding of the topology of brown food webs. Alternatively, it has been argued that conservation policy should aim at preserving species that contribute most to phylogenetic diversity^75^. This last point is particularly relevant for soil microbial systems, for which this information will typically be available due to sequencing. It may be more feasible to identify and protect fragile belowground habitats (for example, where multiple keystone taxa are under threat) through preserving soil structure^76^ and maintaining patterns of succession^77^, rather than working out separate conservation practices for individual species. Once fragile belowground habitats are identified, the challenge will be to address the ‘macrobial bias', the fact that ecosystems with little conservation value for macroscopic organisms are routinely neglected from a conservation point-of-view^78^. This bias is largely due to the fact that non-macroscopic organisms have limited broader appeal. Conservation initiatives for belowground diversity thus need to be well-planned and articulated in sufficiently compelling and accessible ways.

Extinctions in nature are unavoidable. It is also well-established that extinctions represent an ecological opportunity for surviving organisms and are often followed by high speciation rates when viewed over longer time scales^79^. Evolution of microbes can be relatively fast^80^ and species evolving in a changing world, if they evolve fast enough, may be well-equipped to cope with the new challenges. In fact, extinctions of microbes, at present mostly hidden to us, may be far more common than for macroscopic organisms given the ‘long tail' of rare microbial populations^28^. From a basic science perspective, we can turn this to our advantage: soil microbes may actually represent good model systems with which to observe and compare local extinction events occurring in real time and even allow some direct experimentation with the conditions inducing them. There are a number of questions that can be addressed such as how common secondary extinctions are and how fast subsequent adaptive radiation can be. These are phenomena that are difficult to assess for larger organisms, and microbial systems (and particularly belowground microbial systems) could be as instructive as they have been in other fields of ecology^81^. For example, microbial organisms can offer the unique opportunity of studying the conditions under which the ability to survive under severe stress may influence extinction trajectories. Harnessing these opportunities, however, hinges on the development of methods for detection of recently evolved organisms in a community context (Box 3).

### Perspective and future directions

Sophistication of molecular techniques has fostered progress in understanding belowground food webs, and has uncovered a biosphere potentially subject to extinction risk. It is time to make use of this newly acquired knowledge to understand risks associated with survival of belowground organisms, and the prospective impacts on ecosystem functioning. Devising extinction models specific for soil biota is becoming ever more relevant as soil biota play a large role in sustainable ecosystem management; we need to make certain that collection of data is accompanied by the development of pertinent theory for maximum progress in this key area of extinction ecology^82^.

The entire venture could be outlined as a three-step process. First, communicate the importance of belowground biota for ecosystem-services sustainability to aboveground extinction ecologists. This will generate the required momentum for the field to progress and bridge the gap between applied ecologists and theoreticians. Next, adapt existing extinction frameworks to belowground taxa. This is an obvious way to combine existing knowledge with the particular nature of belowground habitats. Finally, test the relative predictive accuracy of the modelling tools that subsequently emerge. This can act as a filtering mechanism for a new generation of extinction models focused on belowground biota. These are all important steps and the most appropriate time to start working on them is now.

## Additional information

**How to cite this article**: Veresoglou, S. D. *et al*. Extinction risk of soil biota. *Nat. Commun.* 6:8862 doi: 10.1038/ncomms9862 (2015).

## Extinction science—Important milestones

The world of extinct organisms opened in the 1600s with the first modern discovery of dinosaur bones^85^, and Niels Stensen's^86^ recognition of ‘tongue stones' to be fossilized shark's teeth . This was also the century of the last sighting of the dodo, though the significance of this fact was not appreciated at the time. Another century passed before Cuvier^87^ showed that mammoths were not the same species as either the Indian or African elephants, but entirely different species. Cuvier^87^ also wrote ‘All of these facts, consistent among themselves, and not opposed by any report, seem to me to prove the existence of a world previous to ours, destroyed by some kind of catastrophe.' Cuvier's^87^ landmark work not only established the reality of extinct creatures but also raised the idea of mass extinctions.

Darwin's theory of evolution by natural selection^88^ argued that the process of extinction was not only real and widespread but was an unavoidable part of the forge of life itself^79^. Natural selection shaped the ecological community through the extinction of some species and the survival of others. On the wheel of evolution, some species could even cause the extinction of others through competition, predation or parasitism^88^.

We tend to think of ‘natural extinctions' as happening in the distance of evolutionary time, and of ‘anthropogenic extinctions' happening here and now. But it is not that simple. Although humans have been around for a million years or more, it was overlooked that we might ourselves be involved in the process of extinction in prehistory. Then Martin^89^ argued that humans, not climate change, were the main driver of the end-Pleistocene extinction. Since then, further evidence of anthropogenic extinctions in the Holocene has come to light, suggesting that the trail of human impact may stretch deep into prehistory^90^. Meanwhile, other research was showing that natural extinctions are not so remote. Van Valen^91^ showed that genus lifetime was 35 million years on average, with the average species lasting about 10 million years. With millions of species on earth, we can thus expect on average one natural extinction to occur every decade, or even every year. Although recent work by Pimm and coworkers^92^ has reduced this rate, it still remains clear that natural extinctions are not limited to deep time, but are close and observable phenomenon^93^.

It is not easy to trace the emergence of a sense of human responsibility towards the natural world, since there have been prophetic voices throughout history. However the establishment of the first major national park, Yellowstone in 1872, and the founding of the Sierra Club in 1892 are important milestones in the emergence of conservation as a political force. In the following century, the establishment of the IUCN Red List in 1963 expressed this explicitly for extinction. The modern concept of an ‘extinction crisis' may be linked to the publication of Myers'^94^ ‘Sinking Ark' in 1979, whereby anthropogenic extinctions were thought to number tens per year until Myers used the species area relation (SAR) to show that the real dimension of the extinction crisis was more like thousands per year .

These large extinctions predicted by Myers^94^ have not been observed^95^. Why is this? The SAR is not wrong: the biodiversity of a rainforest cannot be hosted in an acre plot. However, the SAR is an equilibrium relation while extinctions form part of a dynamic process that takes time. As shown in the study by Diamond^6^, biodiversity may persist, in defiance of the SAR, in a ‘supersaturated' state for thousands of years. All the extinctions will eventually happen, constituting an ‘extinction debt'^35^.

Extinction for soil organisms was overlooked partly because soil organisms are less visible and partly because, being dominated by microorganisms, they have been assumed to be extinction-proof because of the ‘everything is everywhere' assumption. However, it is clear that over long periods of geological time soil microbes do go extinct^37^,^39^. Thus, it is clear that soil organisms, even the microbial ones, are also affected by the extinction crisis.

## Representative examples of local extinctions or significant population declines of belowground organisms

We are aware from studies aiming at resurrecting extinct microbes that some organisms which existed for several million years are only distantly related to existing species^37^. It thus appears that even microbial organisms can go extinct although it is unclear how often such events happen. Natural causes have also mediated a massive local extinction of earthworms. Wisconsin glaciers in 9000 BC are believed to have eradicated earthworms from North America^96^. Recolonization is still in progress, with pervasive effects on ecosystems, and many areas have been invaded by the exotic species that followed European settlers. At smaller scales, local extinctions have been widely observed, for example, for wood-decomposing fungi^20^, which are subject to meta-community dynamics, including local extinctions, due to the ephemeral nature of the wood substrate.

Agricultural components and global change are conspicuous drivers of extinctions aboveground^94^. It appears that belowground anthropogenic activities related to global change and agriculture are having comparable effects but the aspects of these changes that induce extinctions may differ. Elevated CO_2_ and ozone, for example, are expected to be relatively insignificant drivers compared with temperature changes, nitrogen deposition, droughts and other extreme events. However, elevated atmospheric CO_2_ has been shown in the field to have indirect effects on the extinction ecology of nematodes. Niklaus *et al*.^45^ observed reduced soil aggregation levels following exposure to elevated CO_2_. This in turn led to corresponding decreases in soil pore diameters and possibly to the local extinction of larger-diameter nematodes^45^. These nematodes essentially suffered a loss of habitat. Studying the effects of land use on extinction ecology dynamics may be more straightforward, since manipulations do not generally require sophisticated equipment and the response time of the ecosystem is shorter. This has resulted in a wealth of examples with regard to agriculture-related causes.

In a study aimed at reconstructing the soil microbial ecology of North American tallgrass prairie soils, bacteria of the phylum Verrucomicrobia were found to dominate relict prairie, whereas members of this group were considerably less abundant in soils subject to intense agricultural management^48^. Sebacinales, a group of Basidiomycota fungi, were present in organically managed farms included in a survey, but could not be detected with 454-sequencing in conventionally managed farms across Switzerland, indicating that this group suffers large declines with more intensive soil management^49^. Finally, irrigation resulted in local extinctions of the earthworm species *Aporrectodea longa* in farms in New Zealand^47^.

A final category of interest for extinction ecologists are invasion-related extinctions. The most well-documented example of a belowground abundance decline induced by an invasion is that of the exotic earthworm *Aporrectodea trapezoids.* In the Palouse region in United States, invasion of prairie remnants by the exotic earthworm *Aporrectodea trapezoides* has been reported to have brought local earthworm species to the brink of extinction^97^. For fungi invasion of *Hymenoscyphus pseudoalbidus* in Europe, a causal agent of ash dieback, led to a massive decline in the abundance of the phylogenetically related decomposer fungus *H. albidus*^98^ over a period of ∼25 years.

## Tools for studying extinctions in soil

Documenting an extinction in nature represents a major challenge. Contrary to describing new species where finding a single individual may be enough, for an extinction there needs to be unequivocal evidence that no more individuals of the species remain. Even for macroscopic aboveground organisms it is not uncommon that species regarded as having gone extinct are rediscovered. Rather than studying global extinctions directly, a way to proceed in soil could involve using theory to determine rates of local extinctions that might lead (from a meta-community perspective) to local extinctions becoming global. Soil extinction ecology will in general need to rely more heavily on modelling approaches because monitoring is more difficult belowground and because belowground landscapes are more challenging to visualize.

Different approaches need to be brought to bear on the study of local extinctions of microbes and non-microbial soil biota. The obvious distinguishing feature between microbes and non-microbial soil biota is size. As a result, defining what represents a local community for these two groups of organisms may be very different; and this definition of ‘local' may scale up with size depending on the relative ability of these two groups of organisms to interact with their environment.

We commonly implement DNA-based techniques to identify microbes. These have the advantage that relative to the size of the organisms, the amount of habitat sampled is sufficiently large compared with the size of the organisms. They have, however, the downside that single individuals, particularly of bacterial origin, are hard to detect. A way to manage this challenge is through implementing a two-step approach. First, through the use of deep-sequencing, rare microbes could be identified^28^. Subsequently, for a subset of these rare taxa, specific probes can be designed and the dynamics (that is, extinction trajectories) of single molecules representative of certain phylotypes can be studied^99^. In addition it requires much thought to identify evolutionarily independent lineages, based on molecular data, which are the actual target of the study of extinction ecologists, given the widely appreciated difficulty in defining what a species in microbes is. By contrast, for non-microbial soil organisms, such as nematodes or microarthropods, the main challenge is in effectively screening sufficiently large volumes of habitat simultaneously for multiple organisms. A way to achieve this could be through scaling up soil DNA isolation techniques from the existing ∼10-g maximum to volumes that exceed a litre. Simultaneously it is important for taxonomists to work towards linking species descriptions, when they are available, to molecular data^100^.
